# Health Implications of Diverse Visions of Urban Spaces: Bridging the Formal-Informal Divide

**DOI:** 10.3389/fpubh.2019.00239

**Published:** 2019-09-02

**Authors:** Ritu Priya, Ranvir Singh, Sayan Das

**Affiliations:** ^1^Centre of Social Medicine and Community Health, Jawaharlal Nehru University, New Delhi, India; ^2^Health Swaraaj Samvaad Group, South Asian Dialogues on Ecological Democracy, New Delhi, India; ^3^Department of Social Work, Central University of Jammu, Jammu, India

**Keywords:** urban health, urban planning, urban health systems planning, informality, urban inequalities, medical pluralism, health seeking behaviours, politics of knowledge

## Abstract

In the past 200 years, urban spaces have been imagined as neatly laid out, well-planned, sanitised and civilised places of dense human habitation with regulated economic activity, where political power, financial capital, the frontiers of knowledge and technology thrive. This has been the urban planners dream, even while it does not reflect the full reality, whether of cities in the LMICs or the HICs. In the face of such homogenising visions arising from Euro-American models, formal urban systems fail to provide adequately for residents' needs, who then carve out their own resources and processes for meeting them, largely within the domain of urban “informality.” While large part of literature presents urban informality as reflected in the slum, others have shown how it is found in relation to all classes (1). The concept of informality has largely been applied to the core dimensions of economic life of the city. Applied to people's “ways of life,” intermingling of the formal and informal becomes distinctly evident in everyday practices in locations such as the peri-urban, and in activities such as health care. This paper opens up the sphere of health care for urban planning that has, in recent decades, left it largely untouched. It uses data from a rapid assessment of health seeking behaviour of three socioeconomic groups—the middle class, slum-dwellers, and homeless— in Delhi, the capital city of India. The findings, relevant beyond the specific location, reveal that people of all sections resort to myriad informal arrangements for their health care, challenging the dominant connotation of the formal-informal denoting a legitimate-illegitimate dichotomy. This provides potential directions to bridge the formal-informal divide, to re-configure urban planning towards more sustainable futures with plural visions of land use and urban greening for healthier urban conditions and for health care provisioning. The analysis posits that, besides the economic and political relations shaping the formal and informal, the politics of knowledge must be factored in if the informal has to be adequately understood for building sustainable futures.

## Formality, Homogenisation and Centralisation Vs. Informality, Plurality and Decentralisation in Urban Planning

While cities have been the locus of urbanisation, over the past century the urban mode of life has penetrated much deeper, changing everything in its wake. The benefits and opportunities have no doubt been astounding, reflected in the rising longevity of human life across the globe. But no less have been the challenges, including environmental degradation and loss of well-being. Having a higher density of population, higher level of consumption and higher number of industries, urban spaces usually exert a greater pressure on natural resources, frequently leading to greater environmental degradation. Cities account for 75% of energy consumption, more than 60% of greenhouse gas emissions, and 67% of the waste generated (2). South Asian countries present with even more complicated challenges, given their histories of colonisation, underdevelopment, and neglect of these issues in urban planning. Cities run chronically short of water, but their roads routinely get submerged in the rains. High levels of unemployment make people clamour for precarious jobs in industries that pollute air, water and soil, endangering life, and health. As they endanger the environs and economy of the rural areas, the cities also result in enhanced rural-urban migration and further increase in urban densities and needs. Inequalities and insecurities have come to define urban life. Though recognised as major urban problems that are often severer than in rural areas, inequalities in socioeconomic status and consumption, access to services, and in being recognised as legitimate members of the space, remain largely unaddressed or are typically dealt within silos in urban planning. Despite urban populations having better health status indicators on aggregate than the rural and higher density of doctors and medical institutions than in rural areas, health indicators of the urban poor are often not better than of the rural and access to affordable and quality healthcare continues to be severely restricted (3, 4).

A large part of the problem seems to lie in the tunnelled vision of urban planning by the state. Over the past century, with colonial hegemony affecting all spheres, the Euro-American model has provided the imagination of the urban landscape and its planned development world-wide. The local historical social and infrastructural specificities, of course, then bring diversity in its translation on the ground. This has been well-demonstrated in land use planning, housing, water and sanitation systems (5, 6). Planning for catering to urban populations has generally meant formal public services by municipal and other agencies even while land use is decided by technocratic Master Plans and builders' lobbies. In low-and-middle income country (LMIC) contexts, the capital intensive, centralised models of, e.g., water supply and waste disposal, always remained short of demand and resulted in a never-ending chase of targets and counting (and often under-counting) of under-served/un-served population segments. “Informal” mechanisms were adopted by both, the public agencies and the private builders and individuals to meet such basic needs.

Much literature has emerged in recent years on urban “informality,” contrasted with the planners' dream of the manicured formal city. Some scholars view informality as the underbelly of an inequitous development path where large sections of people attempt to find informal means of meeting their basic needs (7, 8). Buoyed by scholarship and experiences from third world cities, urban informality has become an important lens to capture the nuances of urban life, from being a “driver of growth” in De Soto's neoliberal framework, to a liminal space between the structural and everyday practices (9–11). However, other scholars present informality as an integral part of the “planned city” in a grossly unequal social reality, as not restricted to the marginalised, but also part of the way of life of the better off and even evident in the developmental actions of the state, where it flouts it is own master plans, rules, and regulations (1).

In this paper we examine urban informality related to health through a brief discussion on the implications of peri-urban areas, and to health care service utilisation through empirical data from Delhi, the capital city of India. We hope to demonstrate how crucial recognising the informal is in attempting to build people-centred and sustainable futures through planned development.

## Urban Informality

Challenging the dominant Euro American perspectives on urbanism, such as the “modernist” perspective of the Chicago school or the post-modern urbanism of the Los Angeles school, urban informality has emerged as an exciting analytical framework from the experience of the third world cities (12). While beginnings of the informality discourse can be traced back to emergence of the economic concept of the “informal sector” in the 1970s, articulating the movement of labour to the cities in the preceding decades, informality, now as an analytical lens, has opened up newer ways of understanding the mode of the production of space in the urban context. Hart's definition (1973), “Formal incomes came from regulated economic activities and ‘informal' incomes, both legal and illegal, lay beyond the scope of regulation” (13) reflects that “regulated” or “unregulated” is the demarcating line between the formal and informal. Therefore, Kanbur (7) submits that informality can be examined only in relation to particular legislation and regulations.

However, a large body of scholarship from the third world has contested this idea of the informal as unregulated sphere, outside the purview of the state and a refuge of only the poor and marginalised. Scholars like Alsayyad and Roy have shown how governments themselves actively produce informality, by facilitating practices and land use which are contrary to their own regulations and plans. Roy cites the process of rapid peri-urbanisation as an example of informalised process that despite transgressing official plans and norms, often receives tacit sanction from the state. She goes on to identify informality as an idiom of urbanisation in India, as in other countries (10). For example, formal land use planning and regulation in India regularly push the poor outside the formal system, but later accommodate them flouting the same regulations and policies. This gets more pronounced with the inclusion of service provisioning, such as water and waste management, within the domain of informality. Due to incomplete urban infrastructure development, informal networks have been observed to be operating in cities and peri-urban areas to ensure water supply. The formal waste disposal system is vastly supplemented by the informal system of rag pickers and other informal agents. Use of waste water for agriculture in peri-urban areas is well-documented in Indian and other Asian cities as an informal means of waste water disposal and irrigation (10, 14). Hence, informality, contrary to the idea of an unregulated domain, has been argued to represent constant negotiations between various forms of “extra-legal, social, and discursive regulation (10).”

Further, contending the idea that informal cities only represent domains of survival for the urban poor, scholars have highlighted the ingenuous ways in which informality stretches formal limits, opening up newer possibilities for sustainable urban planning. The fluid informal city foregrounds local wisdom in the modern world in opposition to the attempts of its erasure (15). Informality makes visible the ways in which the urban “informals” shape the city fundamentally through their everyday acts of resistance and the processes that negotiate the formal and the informal. Thus, questioning the state centred definition of formality and informality as well as the strict dichotomy in urban configurations, as in the case of land use, water systems, transport systems and so on, they require us to rethink the neat sanitised image of the planned urban space and its urbane civility (9–11).

### Informality as “the Other” Way of Life

Regulatory frameworks related to collective resources such as land and employment play a pre-eminent role in designating entitlements and property relations in urban areas. Healthcare lying in the sphere of “the personal that is political” is somewhat different. The health care industry is part of the formal regulated sector, as represented in modern hospitals and health centres, modern health care professionals, pharmaceuticals, and insurance agencies in the public and private sectors (even if the regulation is often merely notional in third world settings, and often leads to increasing costs and exclusions). However, a large segment of health care that is undertaken in homes and communities, with traditional and new evolving knowledge in addition to the modern conventional providing the basis, lies outside this formal sector and forms the informal healthcare. Since health care includes “ways of life” and application of specialised knowledge, state support and regulation of only a segment of both these makes what is left out, the “unregulated,” seem “illegitimate.” The plurality of knowledge traditions in health care, included in both the formal and informal segments in countries such as India and China, suffers as the traditions with ontology and epistemology differing from that of modern bio-medicine, get delegitimised over the years. While the codified traditions that have adapted to modern institutional forms, are officially recognised and regulated, the officially unrecognised traditional folk practices and practitioners undergo processes of decline. Yet their use, renewal, and innovation continues in efforts to adapt to the challenges of changing conditions (16). While their practice continues, they are pushed to the background in public discourse, propagating perceptions that link them to cultures of ignorance of the uneducated poor and rural.

In the context where urban spaces include a large component of the rural-urban interface (whether as tracts of agriculture that is conventionally viewed as rural activity or by the immigration of rural populations into urban areas), other seemingly rural elements also acquire such an illegitimate characteristic. Studies across third world cities show that peri-urban areas host large concentrations of the rural immigrants in search of livelihoods. In such areas agricultural activity and animal husbandry attracts the poorest and the women, allowing them to find their feet in the urban context with more familiar economic activities and lifestyle. They also provide several other innovative possibilities for urban resilience and sustainability. For instance, peri-urban agriculture has been documented across Asian cities as an informal mechanism devised by local communities to deal with problems generated by an incomplete shift from rural to urban: of mixed land use, unsatisfactory occupational mobility of the local population, domestic waste water disposal, and an influx of rural migrants. Studies show how it allows for retention of green spaces and ecosystem services that provide for increased incomes of the most vulnerable social segments (the poor, women and migrants), food security and waste management (17). However, this urban/peri-urban agriculture tends to be obliterated from public view and not factored into urban plans as a provision for urban green spaces as well as livelihoods, poverty alleviation, food security and the like. In fact, as documented, urban agriculture is completely outside the ambit of the suburban aspirational middle class's vision of an urban area (18).

Simultaneously, the peri-urban space acquires an ambiguous administrative identity, and tends to be left out of public services and amenities. The rural agencies withdraw as the area gets declared to be within the urban boundary, and the urban municipalities take time to reach it since it is peripheral and last priority for distribution of scarce resources. However, since it contains a continuity with traditional ways of life and at least some elements of the eco-system, the possibility of practices such as of food production and herbal medicine are preserved more than in the urban core. Thus, “informality” in itself can be a reflection of deprivation and exploitative relations, or an opportunity for assertion of agency and innovation.

Perceived as spaces with no legitimate identity, peri-urban areas have been conventionally used for dumping the city's waste or its polluting industries for planned sanitisation of the formal city-space. An alternative view is emerging that considers peri-urban spaces as constituting the diversity of urbanisation and providing possible solutions for urban resilience (17, 19, 20). The “urban sprawl” vs. intensive vertical urban infrastructure is central to the debate about urban and regional development models, reflecting the formal architectural urban planning vs. informal land use patterns (21). All these need to be viewed as plural options to be used appropriately as suited to the multiple objectives, needs and contexts even within one city/town/urban agglomeration. Studies on people's innovations that link peri-urban agriculture, waste water disposal, and food security indicate how changing social composition with rural-urban migrants constituting a large part of the residents, livelihood patterns that still depend on eco-system services, and traditional folk practices co-exist. They also demonstrate how the governance structures are completely out of sync with such realities and the many challenges they pose that the formal systems must engage with (14, 17, 20). A holistic view of urban health would do well to examine such systemic issues as central to health systems thinking for improving the health of urban populations.

## Challenges in Urban Health

Public health has been central to urban planning historically and continues to remain a need just as important in the present day urban scenario. Tracing global epidemiological trends of urban health and disease reveals the seriousness of ill-health generation in these most economically endowed areas. Thereby, focusing on urban health behoves us to examine the pathways through which inequality and ecological unsustainability manifest in urban life and shape health and disease. While inequalities permeate every society, the severe lack of resources make matters far more complicated in the LMICs, and attempting to mimic the development models of the high income countries (HICs) has compounded the problem. Nearly a billion people live in slum conditions, with 90% of the urban slums located in the LMICs (22).

The urban space, layered across different socio-political-cultural-economic axes, is mediated differently by different sections of the population, which in turn also shapes their health differently. For instance, Urban Health Resource Centre in New Delhi, India, drawing on data from National Family Health Survey 2005-06, classified urban populations according to their wealth and observed that for almost all indicators on health, healthcare or key social or housing related determinants of health, the poorest quartile fared far worse than the rest of the urban population. Under-five mortality for the poorest quartile was 73 per thousand live births, significantly higher than 42 per thousand live births in the rest of the urban population. In nutrition too the poorest quartile cut a sorry figure with 54% of children being stunted and 47% underweight, whereas the figures were 33% and 26%, respectively for the rest of the urban population. Women receiving at least three antenatal check-ups among the poorest quartile was only 54% compared to 83% for the rest of the population. In terms of living conditions, 81.5% among the poorest quartile lacked access to piped water supply at home and 52.8% did not use a sanitary flush or pit toilet. By contrast, 62% had access to piped water at home and 96% used sanitary toilets among the rest of the urban population (4). Clearly, the inequality is stark, but equally important is the fact that even the non-poor have not achieved health conditions that can be called acceptable in the twenty-first century.

While the urban environment, especially in the slums, offer ground for infectious diseases to flourish, non-communicable diseases (NCDs) are also increasingly laying claim to morbidity and mortality among the urban poor. Precarious employments and exploitative employment relations, poor housing conditions, overcrowding, lack of basic amenities, growing inequalities of consumption lead to deteriorating well-being and rising social strife and violence. Fast-paced life, sedentary work, stressful conditions, unhealthy food habits predispose urban inhabitants of all sections to NCDs like obesity, diabetes, and hypertension. Road traffic accidents, diseases due to environmental pollution are also on the rise. LMIC countries such as India are still in the phase of epidemiological transition where the urban better-off are more affected by the NCDs, but the poorer sections are bearing a disproportionate triple burden—communicable diseases continuing, NCDs emerging and injuries rising rapidly. While socioeconomic vulnerabilities expose them to unhealthy conditions, inadequate, fragmented and expensive health systems rob them of the chance of receiving good quality services. The lack of community outreach of health services in urban areas and poor referral services have only compounded the problem.

The above description of inadequacy of the formal systems to provide for the urban poor, creating conditions of health inequalities is well-documented across the world. Yet, what is often overlooked in discussions of urban health is the fact of the formal systems being limited by their very centralised, mono-solution based, homogenised, bio-medical imagination in catering to human needs. The ignored plural solutions are then resorted to through informal pathways, supporting the existence of a majority of the urban residents. Underlying this problem is international and local political economies, with industries pushing their technological ware (including now the medical and infrastructural “green technologies”) and real estate making its fast buck. But what is less recognised is an epistemological divide that underlies the modern tools of planning— for assessment, validation and regulation of technologies, models, and systems.

India, despite its own experiences in urban planning—for example the city of Shahjahanabad in what is now “Old Delhi”— chose to borrow from western models. Lutyens Delhi in the colonial period or the planned city of Chandigarh post-independence stand testament to that fact. The theoretical assumptions underlying the dominant policy-making knowledge frame, e.g. the Garden Cities of Europe based at least in part on notions of environmental health, led to parks and wide roads at the cost of housing for the poor. Public health needs of the different sections of the population got side lined in the quest for real estate management, often for the benefit of the affluent class. Thus, it has been shown that the inadequacy of urban infrastructure in Independent India, especially for the underprivileged, primarily resulted from the elitist class bias and decontextualised planning of the public administration. Resettling the city's poor from more central locations to the peripheral areas (that had been declared “inhabitable” in the Delhi Master Plan 1962) so as to decongest and beautify the city required official flouting of the masterplan. This led to dismal environmental conditions in these planned colonies of the poor that resulted in endemic water borne diseases and a full blown cholera epidemic in 1988 (23). Adopting decontextualised models from the developed countries meant affording low priority to the common citizens and their specific needs as well as their appropriateness to local contexts. These fail not only the poor but also other residents and frustrate the efforts of sincere administrator-implementors. All of them then flout the rules laid down by the technocratic planning machinery with its globalised elite visions. Given the complex interplay of all these factors, unless the political economy and politics of knowledge is simultaneously acknowledged and issues addressed in a holistic manner, sustainable solutions are unlikely. Whether urban planning, in its theory and in practice, incorporates or can incorporate such an approach is the moot question.

The displacement of polluting industries from Delhi to its peri-urban areas in the 1990s has increased pollution of water, soil, and air that is returning to the city through the food produced in the peri-urban area (14, 18). Now, as the urban dwellers face the consequences of such planning in escalating pollution and public health hazards, *ad hoc* regulations, and drawing natural resources such as water from further afield are implemented as solutions. Given that these do not adequately address the problems generated by the decontextualised vision for urban spaces and their inhabitants, their effects also remain limited and temporary at best.

Similarly, despite a rich heritage of pluralistic health culture, a monolithic health system was imposed upon the Indian people, centred around conventional biomedicine and its doctor and hospital centred institutional model. The system has resulted in a never-ending chase of targets and counting of under-served/un-served population segments. While it responded to the unmet need for services by incorporating para-medical based health centres in rural areas, for urban areas, the imagination remained fixed on hospital and dispensary based medical services until the 1980s. It was in 1983 that a committee recommended urban primary level services with outreach in the community, and even this has been only partially implemented to date (3).

Implementation of the medical institutional model formally adopted suffered from the imperatives of the choices made, with an increasing delegitimisation of the informal. Besides its being too resource intensive, the lack of adequate priority and funding compounded the resource constraints that did not allow the formal plans to be implemented in full (23). Given that the middle class could afford private healthcare and the poor had little bargaining power, there was little public pressure for a strong public service system. As a result, the public system remained anaemic while private healthcare flourished, hurting the vulnerable the most. While strengthening rural health services received some attention through the National Rural Health Mission (NRHM) in 2005, it's counterpart, the National Urban Health Mission (NUHM) is yet to take off in any substantial manner (24). That people still found ways to survive even in these adverse conditions, albeit not in what are considered the best of ways by the formal system, bears witness to their way of life and support structures that existed before state-planned health care, as well as their sheer determination and ingenuity to adapt to changing times. What often came to their rescue in these difficult situations are the informal arrangements that exist and are constantly evolving.

## The Formal and Informal in Urban Health Care

Literature abounds on plural forms of health care in the rural context in India, with almost an implicit assumption that the informal practitioners and practices are located there because of the gross lack of institutional medical services. At present, with 32% of the Indian population in urban areas, about 80% of registered doctors and 80% hospitals are located here, of both the modern conventional and other formal systems. The registered, 5-year degree holding doctors represent the formal health care practice in public and private hospitals, dispensaries, and clinics.

Formal healthcare in India includes eight knowledge traditions recognised by the state— modern medicine and seven other codified health knowledge systems now officially brought under one acronym—AYUSH (Ayurveda, Yoga and Naturopathy, Unani, Siddha, Sowa-Rigpa and Homoeopathy). Each has its texts, colleges for professional education, research councils, hospitals, and dispensaries. However, this has been an “undemocratic pluralism,” with dominance of conventional biomedicine such that it gets over 90% of the government allocation for health while the other seven get 3% (25).

At the other end of the health care spectrum is the practice of home remedies and promotive practices, folk healers and faith healers in the community, as well as family lineages of practitioners of the recognised systems who have been trained inter-generationally without going to college or obtaining degrees. These constitute the “informal traditional” forms of health care, using “free gifts of nature” or easily accessible materials. Folk and faith healers generally provide care as a form of non-commercial community service.

Between these two ends of the informal to formal spectrum newer forms of informal health care have evolved, such as the practitioners of conventional modern medicine who have not been formally educated or are at best trained as paramedics and AYUSH doctors, called Rural Medical Practitioners (RMPs) by some scholars, colloquially called *jhola chaap daktar*^1^. Chemist shop keepers have also become prescribers, thereby becoming another category of informal modern medicine providers. In addition, people have evolved an informal mechanism of preserving the prescriptions of a formal medical professional to use repeatedly in other episodes of the same or other illnesses, by just using it to buy from the chemist. Some modern medicines have also become part of common knowledge and self-care, bought off the counter, such as paracetamol as an anti-fever medicine. This “folkisation” of conventional medicine can be viewed as a market response generating informal providers to fill the gap created by state generated demand which public services could not adequately fulfil, and/or the adoption of industrial products available in the market into self-care practices. “Folkisation” is the term being coined for diffusion of elements of conventional modern biomedicine into everyday popular practice and varies in form and extent across socio-economic sections and regions. Much literature demonstrates the generalisability of this phenomenon across continents (26). Thus, we use folk medicine for whatever people practice at individual, family, and community level without any form of state legitimisation, with “informal traditional” and “informal conventional modern” indicating the knowledge traditions they draw from. While this seems to collapse the folk and the “informal,” there is another form of informal that is not “folk” but embedded in the formal.

Forms of informality are evident within the formal medical institutions, for instance the widespread use of lesser trained nurses and paramedics in private clinical settings with “training on the job” instead of hiring trained professionals who may be in short supply and have to be paid more. Cutting corners or over-prescribing in patient care by private hospitals for enhancing profits often flout professional clinical guidelines. In the over-crowded public sector institutions, scarce resources or time lead to rule-of-thumb practices that do the same (27). Outreach services are often designed to be conducted by paramedics when they ideally require medical supervision. Thus, the formal private and public services too are composed of a spectrum from the formal to the informal providers and practices.

It is generally assumed that the urban areas are devoid of informal health care or that it exists only in slum areas. However, studies in different cities find that all forms of informality among health care providers exist and thrive in urban areas, as does the utilisation of herbal medicine. We would argue that this spectrum of informality in health care and the overlaps between the formal and informal processes reflects what recent scholarship on urban systems has started theorising in other spheres. Also that they are not restricted to India, but are ubiquitous in their presence in all continents and countries, to varying extent and in varying forms.

Studies conducted in Ahmedabad and Cuttack from India found that in urban areas many people still perceive faith healing as an alternative treatment for psychiatric disorders. Faith healing was available easily and locally, in contrast to psychiatric treatment for which most patients and families had to travel a considerable distance (28, 29). Studies from urban areas in Western Nepal, Riyadh, Brazil had observed that in conditions ranging from childhood illness to asthma to respiratory infections, people used home remedies and folk medicine. Knowledge on home remedies was found to be usually inherited from mothers, grandparents, relatives and neighbours (30–32). González-Stuart's (33) study in Mexico's third largest city Monterrey, and Ranjikar and Rajbhandary's in urban areas of Kathmandu valley in Nepal found that despite the widespread use of modern pharmaceuticals and the availability of mainstream medicine in the city, many people still rely on traditional healers and also use medicinal plants to treat diverse variety of ailments (33, 34).

Thus, health care planning for the urban setup, just as urban planning itself, needs to account for the wide spectrum of needs and diverse activities that can cater to them. Mere service provisioning of the conventional type or administrative enhancement of the dominant model of services, out of touch with people's contexts, is unlikely to meet the healthcare needs of the population and for all its segments. People's health seeking and health creating practices are shaped by the myriad of identities they embody. Instead of ironing out such plurality the state needs to address the varied needs and practices of populations.

In India, as in most parts of the world, this, unfortunately, has not been the case. The state's planning for healthcare has completely ignored the traditional community, family and individual based health care. The codified systems that have been officially recognised have a large network of educational, service delivery and production institutions in both the public and private sectors, including a separate Ministry of AYUSH. Folk health knowledge and practices, however, still remain largely bereft of state support. Devoid of the state's patronage, these non-state, non-modern knowledges, and practices have only been able to sustain because of their continuing utilisation, the experience of benefit., and continuing trust that people have placed in them.

### Health Seeking Behaviour Across Three Different Population Groups in Delhi

If urban healthcare planning wants to be truly informed by people's practices, the health seeking behaviour of people relating to both the formal and informal health services need to be examined, keeping in mind its dynamicity and evolving nature. While there have been several studies exploring urban health seeking in the formal health system and fewer in the informal segment, the continuum of care seeking for different sections of population has rarely been explored in the urban context of India. National household level data gathering on health and health care is conducted by several agencies in multiple forms, such as decadal surveys by the National Sample Survey Organisation (35) and the National Family Health Survey (36). While the former categorises treatment sought by any source other than formally trained professionals as “untreated,” the latter documents traditional medicine use only for a few selected conditions as part of a larger survey for health related indicators. Thereby, with extremely limited health seeking behaviour being captured, there has been an invisibilisation of the informal sources of health care by official and public health macro data sets. However, evidence of informal healthcare is widely available from anthropological research and some health systems research studies that demonstrate their pervasive presence across the country, as in a large number of other countries as well (37, 38). In order to begin filling these gaps in information from urban areas, a rapid assessment of health seeking behaviour of different socioeconomic groups was undertaken in 2013-14 in the metropolis of Delhi, the capital city of India.

The methodology adopted for the study was of a rapid survey across three socio-economic classes, the middle-class, the poor, and the homeless. Residential areas of these socio-economic groups were purposively selected, which included a middle-class colony, a slum area and an area with large number of homeless persons. Systematic random sampling was adopted for sampling at the household level in the purposively selected middle class and slum colonies. For the homeless, convenience and snow ball sampling had to be used as they didn't live regularly in any specific location. In total 125 respondents were interviewed (Middle Class−40, Poor Slum Residents−40, and Homeless−45). Majority of the respondents were middle aged women, with diversity in the sampled middle class respondents' educational qualifications while the majority of sampled poor slum residents and homeless were illiterate. A semi-structured interview schedule was used as tool of data collection which was designed to elicit knowledge of all forms of health practices in daily living, including the modern, and traditional modes of care. Previous experience of research among rural and urban communities had shown the importance of the investigator communicating a non-judgemental position on “informal knowledge,” and creating a conversation that encourages respondents to speak about it. Open-ended questions were asked in such a sequence that they allowed the “delegitimised” traditional practice and knowledge to be acknowledged and reported as health seeking actions. The first questions were about what plants and food items the respondent knew had medicinal value and for what health conditions. No checklists were provided from the researcher's side. The information generated reflects the respondent's knowledge of medicinal value of food items in 46 health conditions and plants in 51 health conditions that they themselves cited. One hundred two of the respondents reported any knowledge of food items in health conditions while 113 reported knowledge of medicinal properties of plants. The next set of questions were about their healthcare seeking practice in specific illness events in the past 15 days and over the past 1 year. Given multiple responses from each respondent, the total number of illness episodes for which the respondents sought various modes of healthcare, from home to the outer world, came to 1,114 (Middle class: 327, Poor slum residents: 411, Homeless: 376). Finally, a question was asked about their history of ever visitation to the range of health care providers.

The health care activities reported exhibited a wide range of informal arrangements. Starting with self-care at home (consuming healthy and balanced diets, to using diet change and home remedies or self-medication in minor departures from health), it extended to involve folk healers (herbalists, bone setters, dais, vaids etc.) and faith healers as informal practitioners of traditional systems of medicine available at the level of the community. The chemists and “*jhola chaap*” practitioners constituted the informal providers of conventional medical care. Resort to the formal health system constituted of public or private services of conventional medicine and in small measure to the recognised traditional knowledge systems. For a majority of health issues, home remedies were observed to be the first resort.

**Figure 1:**
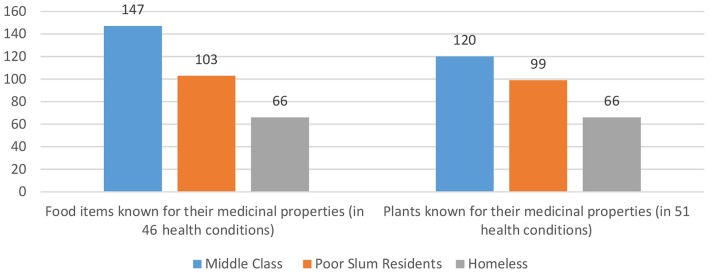
Number of food items and plants used in different health conditions in different socio-economic classes (multiple responses).

Despite the expected erosion of such knowledge within households over the years in the rural and urban populations, the survey found that the general breadth of knowledge on the medicinal properties of food items and medicinal plants was commendably rich. The survey documented that the sampled population is well aware of medicinal properties of food items, 115 of them being reported by respondents to address 46 different health conditions. The survey also listed a total of 91 medicinal plants and their medicinal properties that were known to address 51 different health conditions. Of these food items and medicinal plants, the greatest number was reported by the middle class, then the slum residents and least by the homeless (Figure 1).

When the home remedies or diet change alone did not prove sufficient, people usually resorted to other modes of care in different combinations. The survey traced illness in the last 1 month for this movement of healthcare seeking from home to the outer sources. It was observed that across the three different study groups, while about 15% of illness episodes were dealt with by home remedies and diet change alone, a larger section of care seeking involved home remedies and/or diet change in combination with care from outside home (36.44%). This means altogether home remedies and diet change, alone or in combination with other modes of care, constituted the largest proportion of care seeking behaviour (51.16%). This was 60% for the middle class and poor, while only about 33% for the homeless (whose homelessness actually makes ‘home remedies' a misnomer).

The specifics of treatment sought outside the home showed a multiplicity of patterns of resort, depending on the problem and their collective experience of various forms of treatment for it. For a case of diarrhoea in a child, the treatment would begin with dietary restrictions and home-made oral fluids, quickly move to accessing informal modern treatment, and upon that failing, go to a formal medical provider/institution. In case of arthritis, however, the sequence may lead from home remedies to traditional folk healers and much later to the formal, traditional, or modern.

The three socio-economic groups taken together reveal that, informal health care was resorted to in 67% of illness episodes. This included home remedies and diet change (14.72%), traditional informal (14.18%), and modern informal (38.24%). Disaggregating the data further shows that the modern formal system (public and private) was used most by the middle class (in 72.78% of their illness episodes), followed by much lesser utilisation by the poor households (14.84% of episodes) and the homeless (14.10% of episodes) (**Figure 3**).

**Figure 2:**
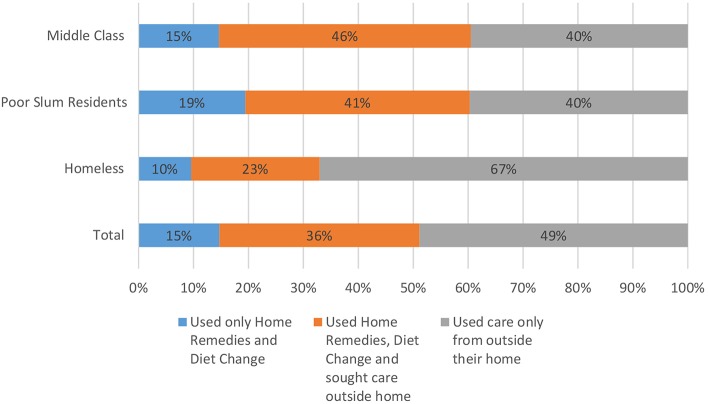
Health care seeking from home to outer world.

**Figure 3:**
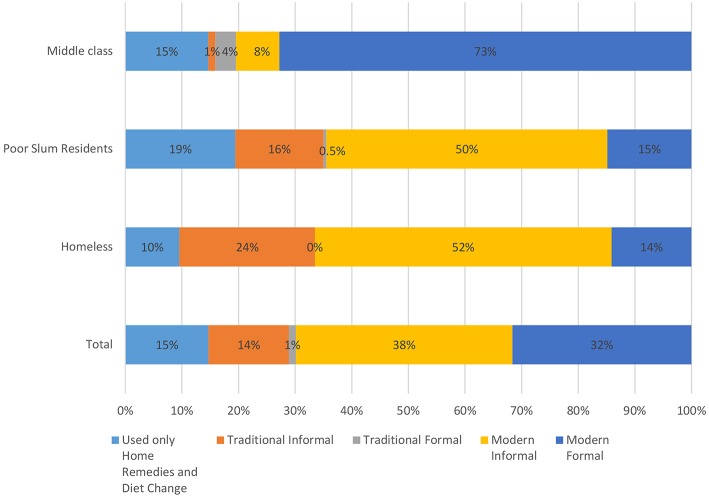
Utilisation of different forms of health care in and outside home. Traditional Informal include, folk healer, faith healer and vaid; Traditional Formal include, AYUSH clinics and dispensaries; Modern Informal include, *Jhola chhaap daktar* and chemist; Modern Formal include, MBBS Doctor/Nurse clinics and nursing homes, allopathic dispensaries, health centres, and hospitals.

The data, although from a small sample, underscores that common people are rational enough to seek out different modes of care when home-based care is insufficient. It is also possible that there is more resort beyond the home than necessary due to the delegitimisation of the folk practices and the over-medicalisation of health, but that is only hypothetical and remains for another study to examine. Clearly, they see care seeking as a continuum between the informal and the formal, not in binaries as the state system makes it (Figures 2, 3).

A telling vignette from the field work during the survey illustrates an important dimension of how the informal reflects the possibility of people's control and sense of ownership of the city as against a sense of passive acceptance and falling in line that is expected from the citizens by the dominant top down planning processes.

While conducting the survey among the homeless, one elderly beggar woman who had lost one arm in an accident was a respondent. She was the strong head of a family of three daughters who were all married with children of their own, but back with her due to widowhood of one, battering and desertion by husbands of the other two. She had come to Delhi almost 25 years ago, searching for a livelihood to feed herself and her daughters after her widowhood. For healthcare she had relied almost exclusively on her knowledge of medicinal plants and other natural home remedies. She gesticulated to where she could find the plants in her surroundings even here in the city, what was available behind the bus-stop and for which plant she had to travel a few kilometres to a greener patch, and so on. She narrated how now, they most often take treatment from the local “jhola chhaap daktar.” Upon asking why this shift, she sadly pronounced, “Now we have lost confidence in ourselves.” In her narrative she had acknowledged the value of the daktar's treatment but also indicated that she thought there was unnecessary resort to the doctor as well.

This is the psycho-social value of the informal, that it allows a critical and problem-solving approach with questioning of the dominant formal, even among the most vulnerable segments of society. Rather than encouraging such bottom up reflection and action, and examining the worth of its solutions, “community participation” is sought ritualistically to ease the implementation of plans made by technocrats based on the dominant knowledge of the times.

The data on “ever visiting health service providers” found that of the myriad providers in the study areas, the middle class, expectedly, visited the allopathic doctor/nurse at the private sector the most (65%), followed by the chemist/pharmacy (50%). Almost half of them had also visited Homeopaths (47.5%), and the Ayurveda practitioners/Vaids (37.5%). Most revealing was the finding that 67.5% of the middle class had also resorted to modern informal providers and 45% had sought treatment from informal traditional providers (Table 1).

**Table 1:** Ever visited health service providers.

**Facility/Health service provider**	**Middle class** **(*N* = 40)**	**Poor slum residents** **(*N* = 40)**	**Homeless** **(*N* = 45)**	**Total** **(*N* = 125)**
Folk healers	9 (22.5%)	7 (17.5%)	9 (20%)	25 (20.0%)
Faith healers	9 (22.5%)	36 (90%)	17 (37.7%)	62 (49.6%)
Ayurveda/Vadis	15 (37.5%)	9 (22.5)	13 (28.8%)	37 (29.6%)
Homeopaths	19 (47.5%)	8 (20%)	–	27 (21.6%)
Siddha practitioners/Tibetan medicine	3 (7.5%)	–	–	3 (2.4%)
Unani practitioners/Hakeem	–	4 (10%)	1 (2.2%)	5 (4%)
Government dispensary	11 (27.5%)	31 (77.5%)	18 (40%)	60 (48%)
Government MCH centre	2 (5.0%)	4 (10%)	2 (4.4%)	8 (6.4%)
Government hospital	4 (10.0%)	20 (50%)	17 (37.7%)	41 (32.8%)
RMP/Jhola-chaap/Non MBSS practitioner	7 (17.5%)	17 (42.5%)	13 (28.8%)	37 (29.6%)
Nursing home (private)	9 (22.5%)	4 (10%)	–	13 (10.4%)
Allopathic doctor/nurse (private)	26 (65.0%)	21 (52.5%)	10 (22.2%)	57 (45.6%)
Chemist/pharmacy	20 (50.0%)	16 (40%)	32 (71.1%)	68 (54.4%)
NGO/charitable allopathic dispensary/hospital/mobile clinic	9 (22.5%)	1 (2.5%)	2 (4.4%)	12 (9.6%)

Of the poor slum resident households 77.5% had ever visited the government dispensary and 50% a government hospital, while 52.5% had gone to the formal private care providers. A much larger proportion (82.5%, 42.5% from Jhola-chaap + 40% chemist) had accessed the informal modern services. It was also observed that reliance on faith healing was the most among the poor (90%) due to the proximity of the slum to a place of worship known for its curative powers. The data also indicates that slum dwellers exercised the greatest “choice” in resort to the range of providers, their number of responses being higher than either the middle class or the homeless, the homeless exercised least choice.

Almost all (99.9%) of the homeless group had sought care from the modern informal providers, and 57.7% from informal traditional care. Care sought from the formal private was the least among the homeless (22%).

While lack of access and affordability would certainly explain in part the limited use of the sources of formal healthcare by the poorer sections, the not insignificant use of informal health care by the middle class testifies to its inherent value as perceived by all classes. Strengthening the formal public services would, of course, be of benefit to the urban residents especially the poor. However, recent fledgling initiatives at health systems strengthening are unlikely to have impacted treatment seeking behaviours in our study population since 2013-14 when our study was conducted. Delhi government's recent initiative of setting up “Mohalla Clinics” (Colony clinics) for providing free diagnostics and drugs has a very restricted population coverage and the Ayushman Bharat initiative of the national government providing social insurance to the poorest 40% of population is restricted to covering hospital care, with both still facing problems of implementation and fragmented design (39–41).

It is apparent from the data above that a majority of illness episodes are taken care of by informal mechanisms, while the state plans only for the formal health care. Pluralism allows the urban residents to exercise their agency to choose between use of the home remedies they know and the informal and formal sources of healthcare outside the home, in various combinations as they are able to access or think appropriate. The implications of this for urban health care and planning need to be understood.

## Discussion

Cities encompass multiplicities within themselves, where various concepts of urbanism coalesce and fragment simultaneously to create ever shifting kaleidoscopic arrangements. In the cities of Latin America, Asia and Africa, the situation gets additionally complicated with their histories of colonisation, underdevelopment and the attempts to develop by adopting the expensive and high consumption models of the first world, as well as the presence of a market dominated economy. An exploitative economic system in conjunction with deeply entrenched social hierarchies have created a landscape of precarious employment and perilous living conditions for the urban underclass, all marking the pervasive poverty by the notion of informality. Rapid globalisation, aggressive marketisation and a receding state have further attributed the cities of the third world with the defining characteristics of increasing inequality, rising violence and an unruly politics. While the “informal” urban processes are a response to such inequality in access to basic resources and services, they are also the means of fulfilling human needs of all sections that the formal system is failing to do., What this paper argues is that it is not only an economic inequality, but also the power equations in the politics of knowledge that denies the more local and rural popular epistemologies and ways of life due consideration in the visions for planning urban spaces, “the urban way of life” and urban health care. Informality reflects not only the solutions of low resource settings in urban areas, but also the marginalised epistemologies that resurface even in better resourced settings, and in people's attempts to conserve or continue to practice what they value in their way of life, and in the cultural-moral underpinnings that accompany them. The wide-spread and increasing informal resort to traditional-complementary-and-alternative medicine (TCAM) in HICs is beginning to be formally factored into their health service systems, for instance by medical insurance paying for TCAM services, the teaching about TCAM in medical schools to sensitise under-graduate medical students as well as in specialisations for post-graduate studies. But the prevailing informal traditional knowledge and practices of the urban poor are likely to be lost to them in the prevailing environment in most LMICs. Traditional folk practices continue to be delegitimised, and with increasing “folkisation” of conventional bio-medicine, forms of self-care and informal providers based on use of modern conventional industrially produced medicine replace the more ecologically sensitive understandings of health as a product of the ecosystem and way of life and health care based on natural materials and processes. Implications of these trends need to be examined.

The concept of urban informality that has largely been applied to urban land use as well as to economic activities and livelihoods related to them, has been extended in this paper to the pluralism of health care in people's lives as against the official planning of health care that has ignored such possibilities. The study showed a spectrum of informal and formal-informal sources of health care forming the urban health care system on the ground, even in a metropolis such as Delhi, the capital of an emerging economy. All the sources of formal and informal health care were resorted to by all sections of urban residents, the poor and middle class, in a plural combination of health seeking behaviour varying by socio-economic status. On an aggregate, only 33% of illness episodes led to resort to the formal services (often combined with informal measures) while 67% were dealt with through informal sources of health care. Care seeking for promotion or preservation of health begins from the informal setup of the home across all socio-economic groups. These practices of self or family care, although constituting the largest proportion of healthcare, receive very little attention from the state which arranges all its planning and activities around formal sources of healthcare even when its use is much limited in comparison. Outside care for the poor and the homeless comes mainly from informal health service providers, but they are sought out even by the middle class. Clearly people make choices across the wide spectrum of informal to formal depending on the nature of their ailment and experience with different systems, influenced by their location and belief systems. The exclusivity of what is legitimised as valid knowledge and its practitioners by the state's vision of health care (through official recognition and regulatory mechanisms that mark out the formal in health care with ignoring of all that is outside it), is rarely observed in real life practice.

The way formal urban health care is planned in India carries common attributes with urban planning in general, The models tend to get adopted uncritically from international contexts. While there is a diverse set of health services and practices available in the urban culture of Delhi, the state reserves its support for only institutionalised biomedicine, which has become the standard the world over, or that part of traditional medicine that has moulded itself into the dominant model of doctor-and-institution centred health care. Solely a disease centred approach limits the biomedical system to primarily curative services and that gets reflected in the state supported health services as well. A more democratic and comprehensive approach to health planning in the urban setup would make use of not only the plurality of knowledge and practices available, but also link health with its various other determinants, such as employment conditions for the urban worker, livelihood and food security, living conditions etc. Urban planning, instead, driven by the desire to implement universal solutions, creates an abstract homogenisation of urban life, mostly out of touch with reality of the majority of the citizens. Rather than learning from local wisdom of diverse people and communities, “the formal” attempts to centralise systems of knowledge and of production and application, clearly evident in water supply, sewage and institutionalised healthcare provisioning in the urban areas. This creates inappropriate services and wastage of valuable resources. Being far removed from the local context the models adopted are more aspirational of the “development standards” of the global north rather than mindful of ecological sustainability, economic viability, and social justice.

The observation of widespread resort to informal modern conventional health care in cities, towns and villages has led to the proposal to officially institute 3-year courses for training “community-level accredited practitioners—not full-fledged doctors” who can practice independently and provide basic health care at low cost (42). However, this policy measure has not found favour with the regulatory bodies in the country even when attempted by some states for rural areas (43).

Moreover, the dominant politics of knowledge overlooks, even delegitimises, these alternative sources of knowledge coming from the common people of all sections. Thus, even though the most marginalised of the population primarily depended on informal sources and the public services for their healthcare needs, there's little reflection of that in existing plans that favour promotion of private medical services. People taking care of themselves, with locally available resources, is a reality that is usually ignored in our formal health services planning. The whole articulation is geared only towards medical service provisioning and demand generation for the products of the medical industry, creating dependency rather than facilitating people's own health creating practices.

It must be admitted that folk medicine like any other system of medicine has its own strengths and limitations. While it is mainly used in present times for promotion of health and treatment of common everyday health problems, its value as beneficial for health promotion, prevention, palliation and cure has been well-recognised by pharmacology since the discipline has evolved on the basis of traditional medicinal plant knowledge found especially in the “developing” world. The pharmaceutical industry too has depended on it as the source for leads to identify medically effective molecules for its research and development activities, and continues to do “bio-prospecting” even in the present (44). In recent years, the state has opened up to civil society initiatives attempting some form of recognition of the folk practices and practitioners—for instance, the Indian Public Health Standards set by the Ministry of Health and Family Welfare for rural health services that stipulate cultivating herbal gardens in the compounds of Primary Health Centres and Sub-centres as “desirable” (45, 46). Evolving systems for certification of traditional folk healers such as herbalists, and bonesetters and their practices by the Quality Council of India (a quasi-statal agency) (47) is another initiative relevant to bridging the formal-informal divide. However, even this is targeted at the rural areas, with no acknowledgment of the existence and practice of folk medicine in urban areas. Codified systems other than the modern conventional too are growing in their scope of urban practice and integration into modern health care by adopting modern forms of institutions and regulation. Yet, even this component of the formal in major Asian countries is not factored into most of health systems research or design, either in the Primary Health Care of 1978 or in Universal Health Coverage of the 2000s or the international slogan for attaining the Sustainable Development Goals (48). Even those advocating for addressing the Social Determinants of Health as part of UHC for South Asian cities do not seem to envisage plural forms of health care as a relevant component (49). In contrast, Cuba, a country well-recognised for the large number of doctors it produces and its well-developed modern health services, is, some may think paradoxically, also a pioneer in the scientific research and use of Homeopathy as well as teaching of herbal medicine to school children (50). This seems to embody the healthcare system with what Knutstadt had hypothesised in 1975, that “pluralism of medical beliefs, choices, and therapeutic strategies offer adaptive advantages to health care systems. Instead of producing negative effects, as some proponents of the symbolic unity of cultural systems have led us to believe, cognitive dissonance (multiple and competing health care strategies), at least in the health care system, may well have distinct advantages for biological survival, the resolution of psychosocial tensions, and the evolution of adaptive cultural strategies” (51).

Our study shows the possible benefits of providing urban populations access to traditional medicinal plants by promoting urban forests and herbal gardens in public spaces as well as encouraging cultivation and use of medicinal plants and herbs by all sections. Urban/peri-urban agriculture and cultivation of medicinal plants in public spaces for citizens to access is possible with a vision of urban green spaces that may not be neat or sanitised but are of use in the way of life of all, and especially of the poor and migrant. Learnings can come from initiatives such as undertaken in Cape Town, South Africa that brought together disparate groups who share interests in the city's biodiversity—formal economy, predominately middle class conservation managers and policy makers, and informal economy, predominately working class Rasta herbalists—to develop herbal gardens in public spaces (52).

State can play an important role therefore in reviewing the popular practices and facilitating those that prove beneficial. In terms of strengths beyond biological efficacy, it is important to note that folk traditions are commonly an integral part of the social and physical environment of the communities, making them more conducive for adoption and treatment adherence. Facilitating home remedies which are usually low cost, prepared from locally available materials, and constituting a significant proportion of care seeking, could have not only brought down the cost of care but also taken the load off the formal public services, allowing them to improve their quality of services. In the face of exorbitant rise of medical care cost, increasing incidence of antimicrobial resistance and deaths due to iatrogenesis globally, as well as the polluting nature of the pharmaceutical industry, the traditional systems of medicine and folk medicine do hold promise of contributing to evolving more democratic, plural and sustainable health systems.

An integrated, comprehensive, and ecological planning would have optimised the existing resources and practices in providing people good quality care that is need based and sustainable on a longer term. Epidemiological logic in consonance with people's need and service provider's opinion, should inform decisions regarding provision of institutional care. An earlier study covering rural populations spanning 18 states of India had found that 80% items listed as self-reported household level knowledge of medicinal value of plants and food items was verified and validated to be rational by the codified knowledge and scientific principles of the recognised AYUSH systems (37). Building on urban people's existing knowledge of medicinal properties of food and plants, as demonstrated by the data in this paper, should create an interface of the formal institutional with the informal that remains sensitive to people's agency and innovation (53). Documenting and researching home based remedies, disseminating useful knowledge about them, creating herbal medicinal gardens in urban localities, teaching children in schools about the value of medicinal plants and involving them in their cultivation and use, could go a long way in re-legitimising this knowledge and bringing its benefits to larger numbers. Such measures are rarely resource intensive and due to their dependence on nature, also provide people with a reason to preserve the natural habitat.

The challenge to such folk knowledge and practices that tend to be location specific can pose challenges when practitioners migrate to urban areas. Our study found that there is continuity of the regional flora across rural and urban, which gets eroded as construction and concretisation of open spaces increases. While this problem can be resolved, the issue of folk healers is more complex. The folk healers were traditionally regulated by the norms and ethics of their community of healers. As they migrate to the city, they can get cut off from their community and its regulatory system. That is why wider propagation of the peer assessment based certification mechanisms for folk healers becomes important even in urban areas. Further, their skill upgradation through senior healers would ensure survival of such valuable knowledge and its practice brought to better benefit larger numbers. Inter-disciplinary and trans-disciplinary research could also provide systemic mechanisms to rationally increase the element of self-care and community level care so as to decrease resort to institutional care, thereby decreasing costs and time of both the users and institutional providers. Further, based on studies of the urban poor in different parts of India and a wider literature review, it has been argued that their perceptions of quality of care, understandings of the social determinants of health and ways of dealing with them can provide learnings for more appropriate assessment criteria and designing of health systems (54, 55). In the 1970s, based on Chinese health culture, Kleinman famously depicted the health care system as being composed of three distinct spheres—“the popular” (individual, family and community-based) as the largest, with smaller spheres of “the professional” or formal and “the folk” composed of folk practitioners (51). While finding that this holds true even in the twenty-first century urban context (though we use “folk” to include the popular practices and the non-formal providers), our analysis shows that there is greater complexity within and inter-digitation between them. The relationship between these spheres is therefore better depicted as concentric circles, as in the paper by Ghodajkar et al. (53). This has implications for how the health care system boundaries are to be drawn and how regulatory as well as institutional structures are to deal with the various spheres.

Thereby, the informal can (i) be analysed to understand what features suit people in diverse conditions and build them into system or intervention designs, (ii) demonstrate pathways to sustainability in diverse contexts, and (iii) be analysed for evolving normative principles for planning. All these are valid activities even while they need to be undertaken with the recognition that the informal mechanisms may not always be the “best solutions” of the elite, but responses for coping with situations full of constraints. The constraints themselves have to be recognised as those of societal resources, as relevant considerations for any efforts at sustainable development encompassing all socio-economic sections and thereby questioning the criteria for assessing optional solutions including those of the elite. The interface between the informal and the formal is the junction we are hoping to address for examining and contributing to building sustainable systems.

People's health seeking practices show that there is place for both the formal and informal. The how, where and what of that gets reflected in the choices that people make which vary by specific contexts. Making the process of urban planning people centred should therefore create conditions that empower people's solutions emerging from the ground. This can help capture the complexities of real life, as well as show us how to make the urban environment healthier by ensuring fulfilment of basic needs and promoting resilience (e.g., with promotion of peri-urban agriculture) and the health care system more responsive to people's needs and suitable to local context (e.g., promoting people's access to medicinal plants in public spaces). Sustainable cities, while upholding the three pillars of social equity, economic prosperity and environmental integrity, need to place democratisation of knowledge and people's practices upfront as the fourth pillar. Exploring urban informality can provide us with some clues on how to do that, so as to reflect the diversity within urban areas and provide more sustainable options for organising the high urban densities into more livable spaces for health and well-being of all.

## Data Availability

The datasets generated for this study are available on request to the corresponding author.

## Author Contributions

The empirical study was conceptualised by RP. Methodology and tools prepared by RP and RS. The survey was coordinated and data managed by RS. Data analysis was done jointly by RP and RS. RP and SD jointly conceptualised and wrote the paper based on an earlier draft by RP and RS.

### Conflict of Interest Statement

The authors declare that the research was conducted in the absence of any commercial or financial relationships that could be construed as a potential conflict of interest.

## Footnotes

^1^“*jhola chaap daktar*” refers to the non-MBBS practitioner of conventional medicine who often travel to settlements of the poor and rural, carrying medicines and basic equipment with them, but also includes those who set up clinics in the areas of the poor.
